# TGF‐β signaling in liver metastasis

**DOI:** 10.1002/ctm2.160

**Published:** 2020-11-30

**Authors:** Dieuwke L Marvin, Rosan Heijboer, Peter ten Dijke, Laila Ritsma

**Affiliations:** ^1^ Department of Cell and Chemical Biology and Oncode Institute Leiden University Medical Center Leiden The Netherlands

**Keywords:** cancer, immunotherapy, liver metastasis, targeted therapy, transforming growth factor beta, tumor microenvironment

## Abstract

The presence of liver metastases drastically worsens the prognosis of cancer patients. The liver is the second most prevalent metastatic site in cancer patients, but systemic therapeutic opportunities that target liver metastases are still limited. To aid the discovery of novel treatment options for metastatic liver disease, we provide insight into the cellular and molecular steps required for liver colonization. For successful colonization in the liver, adaptation of tumor cells and surrounding stroma is essential. This includes the formation of a pre‐metastatic niche, the creation of a fibrotic and immune suppressive environment, angiogenesis, and adaptation of tumor cells. We illustrate that transforming growth factor β (TGF‐β) is a central cytokine in all these processes. At last, we devise that future research should focus on TGF‐β inhibitory strategies, especially in combination with immunotherapy. This promising systemic treatment strategy has potential to eliminate distant metastases as the efficacy of immunotherapy will be enhanced.

## Graphical abstract

The liver is a permissive organ for metastasis, but adaptation of both liver environment and tumor cells is necessary for metastatic outgrowth. During liver metastasis formation, the cytokine TGF‐β promotes hepatic colonization by affecting the tumor cells, as well as tumor stroma, vasculature, and immune cells. Targeting TGF‐β, in combination with immunotherapy, has the potential to eliminate liver metastasis.

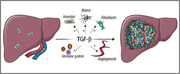

**Abbreviations**
HSC: hepatic stellate cell
aHSC: activated hepatic stellate cell
BMDC: bone marrow derived cell
CAF: cancer associated fibroblasts
CCL: C‐C motif chemokine ligand
CCR1: C‐C chemokine receptor type 1
CRC: colorectal cancer
CTGF: connective tissue growth factor
CXCR4: C‐X‐C chemokine receptor type 4
ECM: extracellular matrix
EMT: epithelial to mesenchymal transition
ERK: extracellular signal‐regulated kinases
FGF: fibroblast growth factor
FOXp3: Forkhead box p3
FSP1: fibroblast‐specific protein
HCC: hepatocellular carcinoma
HGF: hepatocyte growth factor
HIF‐1α: hypoxia inducible factor ‐1alpha
ICAM‐1: intracellular adhesion molecule
IFN‐γ: interferon gamma
IL‐6: interleukin 6
IQGAP1: IQ motif containing GTPase activating protein 1
JNK: c‐Jun N‐terminal kinases
LAP: latency associated peptide
LCC: Lewis lung carcinoma
LOX: lysyl oxidase
LSEC: liver sinusoid endothelial cell
MAPK: mitogen‐activated protein kinase
MDSC: myeloid derived suppressor cell
MIF: macrophage migration inhibitory factor
MMP: matrix metalloprotease
MSC: mesenchymal stem cell
NK‐cell: natural killer cell
PD‐(L)1: programmed cell death (ligand) 1
PDAC: pancreatic ductal adenocarcinoma
PDGF: platelet derived growth factor
PKA: protein kinase A
Rock: rho‐associated coiled‐coil kinase
SDF: stromal derived factor
SMAD: mothers against decapentaplegic homolog
Smurf: smad ubiquitination regulatory factor
Spr1: sphingosine‐1‐phosphate receptor 1
STAT3: signal transducer and activator of transcription
TAM: tumor associated macrophage
TGF‐β: transforming growth factor beta
TRAF: TNF receptor associated factor
TβRI/II: TGF‐β type I/II receptor
VEGF‐A: vascular endothelial growth factor‐A
YAP: yes‐associated protein
α‐SMA: alpha‐smooth muscle actin

## 1 INTRODUCTION

Cancer is one of the leading causes of death worldwide.^1^ The development of cancer metastasis drastically worsens the prognosis of cancer patients and contributes to the majority of cancer related deaths.^2^ Despite many developments in anticancer therapy, metastases remain hard to treat.

**FIGURE 1:**
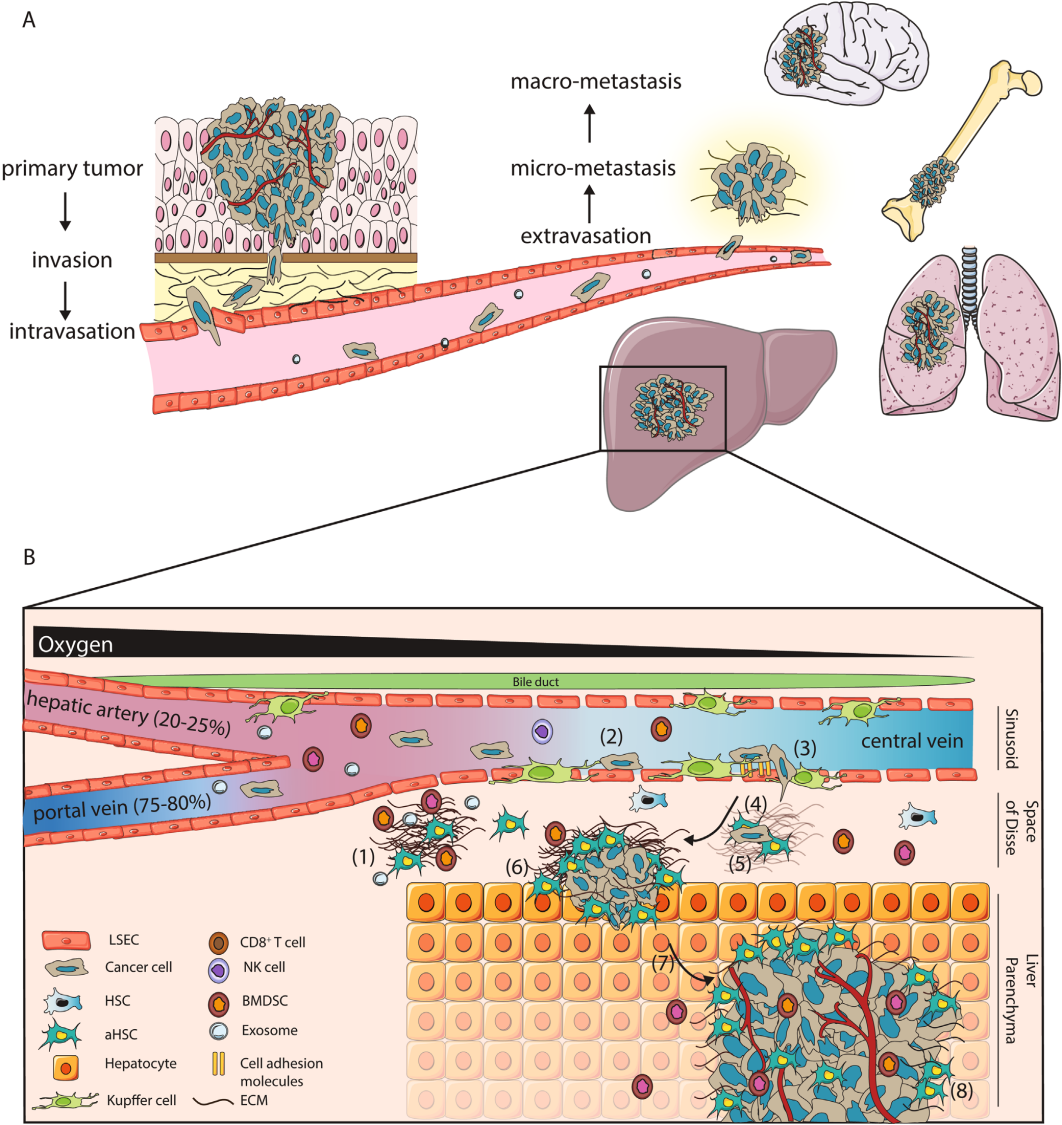
The metastatic cascade and formation of liver metastasis. A, Metastasis is a multistep process starting with invasive cells at the primary tumor site that invade the basement membrane and intravasate into the vasculature. After traveling through the vasculature, tumor cells can extravasate to form micrometastases and eventually macrometastases in secondary tumor sites such as bone, lungs, brain and liver. B, Colonization of the liver can be divided into (at least) eight different steps. Prior to arrival of tumor cells, pre‐metastatic niches can be created to favor tumor cell through secreted factors and extracellular vesicles by primary tumors (1). Tumor cells can arrive in the liver through the portal vein or hepatic artery, where after they arrest in the liver sinusoid (2). Upon extravasation into the Space of Disse (3), niche remodeling (4) is essential for survival and overcoming latency (5). When favorable conditions allow tumor cells to grow out into micrometastasis (6), angiogenesis (7) can allow the formation of macrometastasis (8). Abbreviations: LSEC, liver sinusoid endothelial cell; (a)HSC, (activated) hepatic stellate cell; ECM, extracellular matrix; NK‐cell, natural killer cell; BMDSC, bone marrow derived suppressor cells

The metastatic cascade starts at the primary tumor site, where tumor cells can gain invasive characteristics, allowing penetration of tumor basement membrane and intravasation into the vasculature. Traveling through the vasculature, tumor cells can reach secondary sites, extravasate, and colonize the secondary organ, depicted in Figure 1A.^3^ Previous research has shown that many tumor cells disseminate to distant organs, however, very few survive in distant niches.^4^ As such, colonization can be seen as the bottleneck for metastasis. Adaptation of the tumor cells as well as the microenvironment is essential to overcome this bottleneck.

Sites of metastasis can be influenced by both anatomical and niche characteristics, as proposed by two theories; Ewing's size constraint theory and Paget's seed and soil theory.^5^, ^6^ The liver is one of the most prevalent metastatic sites for many cancers, due to its anatomical location and blood supply through the portal vein and the hepatic artery, as well as its permissive microenvironment.^7^, ^8^ Colonization of the liver can be divided into (at least) eight distinct phases, as shown in Figure 1B, starting with induction of a pre‐metastatic niche prior to tumor cell arrival, to establish liver tropism and promoting later outgrowth of tumor cells (1).^6^, ^9^ Upon arrival, tumor cells arrest in the liver sinusoids or terminal portal venules (2), followed by extravasation into the Space of Disse (3). When tumor cells resist or evade local anticancer immune responses, a supportive niche needs to be created through remodeling the local microenvironment and recruiting stromal cells (4). Adaptation of the local niche is essential for development into the next phases. After a possible latency period (5), tumor cells have to divide to form micrometastases (6). When angiogenesis is successfully induced (7), full blown macrometastases can be formed (8).^8^, ^10^

In order to therapeutically target the bottleneck of metastasis, it is essential to understand the underlying signaling processes that allow for niche adaptation and tumor cell adaptation. The regulatory cytokine transforming growth factor beta (TGF‐β) has been shown to be of key importance in metastasis formation, both in tumor cells and different aspects of the tumor niche.^11^, ^12^ In cancer, TGF‐β can elicit a dual role, suppressing tumor growth in early phases, in contrast to functioning as a tumor promotor in later phases.^12^ In addition, TGF‐β is involved in many processes in epithelial, endothelial, and neural tissues, the immune system, and wound repair.^13^ All TGF‐β family members have multiple functions and the consequence of their activation is highly dependent on the cellular context. TGF‐β has pivotal roles in maintaining homeostasis in the liver and is expressed in various degrees by cells in the liver.^14^, ^15^ For example, TGF‐β aids to liver regeneration, however, high levels of TGF‐β signaling can lead to liver fibrosis.^15^ Because of this complex multifaceted role of TGF‐β in both liver microenvironmental cells and tumor cells, and because of its expression in the liver by multiple cell types, TGF‐β can have a leading role in liver metastasis formation and can pose as a suitable target for anti‐cancer treatment, tackling multiple facets of metastasis formation.

In this review, we will give an overview of the processes involved in liver metastasis and highlight the current knowledge on the contribution of TGF‐β to liver metastasis formation. Recent progress in TGF‐β targeting, especially in combination with immunomodulatory therapy, will be reflected and put in context of the different layers of complexity of TGF‐β signaling during liver metastasis formation. Taking multiple angles of TGF‐β signaling into account, recommendations will be made for future therapeutic intervention of liver metastasis.

## 2 METASTASIS FORMATION IN THE LIVER

### 2.1 The liver is a common site of metastasis for different cancer types

Due to the drainage of venous blood from the abdomen to the portal vein, gastrointestinal cancers such as colorectal and pancreatic cancer most frequently metastasize to the liver.^7^, ^16^ Other cancers regularly presenting liver metastasis are, for example, breast cancer, (uveal) melanomas, pulmonary cancers, and sarcomas.^16^, ^17^, ^18^, ^19^ Surgery aiming for curative resection is the main treatment option for resectable liver metastases.^17^, ^20^, ^21^ Additionally, systemic treatment or local treatments such as ablation and liver perfusion with anticancer treatment can be of benefit.^22^, ^23^ Unfortunately, few patients are eligible for surgical resection, and chances of recurrence can increase after surgery.^24^, ^25^

### 2.2 The liver has a complex structure consisting of specialized cell types

The liver has unique anatomical properties consisting of specialized cells to execute the many functions of the liver (Figure 1B).^26^ In the liver, oxygen rich blood from the hepatic artery is mixed with venous blood from the portal vein in sinusoids, which is surrounded by the Space of Disse.^26^, ^27^ Hepatocytes represent the largest cell population in the liver and are responsible for most metabolic and detoxifying functions together with cholangiocytes, the epithelial layer lining bile ducts. Hepatic stellate cells (HSCs) are present in the Space of Disse in a quiescent form and function as storage for retinol esters in lipid droplets. Local damage signals can lead to HSC activation (aHSC), that is, myofibroblasts characterized by alpha smooth muscle actin (α‐SMA) expression, resulting in HSC proliferation and secretion of different cytokines and extracellular matrix (ECM).^28^ HSCs are the main source of myofibroblasts, however hepatocytes, portal fibroblasts, bone marrow derived fibroblasts, and cholangiocytes can differentiate into myofibroblasts as well.^29^ Specialized liver macrophages, Kupffer cells, represent the majority of the immune cell population in the liver and are located in the sinusoids. Liver sinusoid endothelial cells (LSEC) are specialized to maximize blood flow exchange and contain fenestrae.^26^, ^30^, ^31^ Blood flow is kept low in the sinusoids. The presence of fenestrae and low blood flow, together with the supply of both venous and arterial blood, make the liver anatomically a permissive organ for metastasis. Moreover, the different stromal cell populations in the liver can provide favorable niches by secreting adhesion molecules, cytokines, growth factors, and extracellular matrix (modulating) proteins, promoting tumor cell adhesion, survival, growth, and angiogenesis.

### 2.3 The different phases of liver colonization

Colonization of the liver can be divided in distinct phases (Figure 1B).^10^, ^32^ Dynamic interaction between tumor cells and the local microenvironmental during these phases is key for successful colonization. The role of individual cells in the liver microenvironment during colonization is described in detail by^8^, ^10^ and is summarized below.

#### 2.3.1 Step 1: Pre‐metastatic niche facilitates metastatic outgrowth

Recent research has shown the presence of pre‐metastatic niches that exist at distant sites prior to the formation of metastases.^6^, ^9^, ^33^ Even though the existence of the pre‐metastatic niche is hard to prove in a human setting, it can be recognized as the first step of liver colonization. Primary tumors can secrete cytokines and extracellular vesicles containing, for example, microRNAs, integrins, and cytokines, which can modulate distant niches including the liver, thereby promoting metastatic outgrowth. For example, by promoting fibrosis or recruitment of myeloid or MDSC to the liver.^34^, ^35^ Integrins in exosomes can influence tropism of metastasis through preferential binding to specific organs. For example, integrin β5‐expressing exosomes were found to specifically adhere to Kupffer cells in fibronectin high liver tissue, where they induce upregulation of pro‐migratory and pro‐inflammatory S100 proteins.^6^ Exosome‐derived integrins have also been shown to activate HSCs to induce a pro‐inflammatory environment in the liver, and stimulating stemness and epithelial‐to‐mesenchymal transition (EMT) in later colorectal cancer (CRC) metastases.^36^

In conclusion, the liver niche can be adapted to favor tumor cell survival prior to tumor cell arrival through secretion of cytokines and exosomes by the primary tumor.

#### 2.3.2 Steps 2 and 3: Tumor cell arrest in liver sinusoids and extravasation

Due to the hostile environment of the blood, including hemodynamic forces and immune cell clearance, most disseminated tumor cells will be eliminated in the circulation.^3^ Upon successful arrival of tumor cells in liver sinusoids, cells encounter the first line of defense of the innate immune system of the liver. Kupffer cells can eliminate tumor cells through phagocytosis, which can be promoted by other recruited inflammatory cells, including natural killer (NK) cells.^8^ Local NK cells and CD8^+^ T cells can induce apoptosis of tumor cells through FAS ligand/receptor binding, by forming perforin pores and stimulating granzyme release.^8^, ^37^ Tumor cells can resist or evade local anticancer immune responses through, for example, downregulation of the major histocompatibility complex class 1 or arriving as tumor cell clumps.^38^ Interestingly, when large quantities of tumor cells arrive in the liver, Kupffer cells can switch to a tumor promoting role, secreting different tumor supportive cytokines including hepatocyte growth factor (HGF) and IL‐6.^8^, ^39^

Next to the local immune system, tumor cells encounter LSECs upon their arrival. LSECs are rich in cell adhesion proteins and surface oligosaccharides, facilitating the adherence of tumor cells, and stimulating the survival and migratory capacity of tumor cells.^40^, ^41^ Cytokines, secreted by Kupffer cells as well as by arrested tumor cells, can lead to increased adhesion and extravasation of the tumor cells to the sinusoid endothelium, through induced expression of certain adhesion molecules on LSECs, including E‐selectin, vascular cell adhesion molecule‐1, and intracellular adhesion molecule (ICAM)‐1.^39^, ^42^, ^43^, ^44^, ^45^, ^46^, ^47^ Thus, conditions upon tumor cell arrival in the liver can be modulated to promote tumor cell survival and extravasation into the organ.^39^

#### 2.3.3 Steps 4‐6: Adaptation to local niche leads to micrometastasis formation

Once extravasated, tumor cells arrive in the Space of Disse. Here, a large number of extravasated tumor cells stay in dormancy, although it is not required.^4^ Progression to micrometastasis formation is dependent on the evasion of the immune system and the induction of secretion of ECM (remodeling) proteins, growth factors, and cytokines by myofibroblasts. This will promote tumor cell survival, invasion, growth, and create a favorable immune suppressive environment.^28^, ^29^, ^48^, ^49^ Through tumor‐stroma crosstalk, tumor cells are able to recruit and activate surrounding liver microenvironmental cells into myofibroblasts.^10^, ^28^, ^32^, ^48^, ^50^, ^51^ TGF‐β signaling is key in this crosstalk. Activation of HSCs into aHSCs leads to secretion of cytokines, including platelet derived growth factor (PDGF), HGF, TGF‐β, stromal derived factor (SDF), ECM proteins fibronectin and collagen, and matrix metalloproteases (MMPs).^28^, ^48^, ^49^ This matrix deposition can induce tumor cell growth and could provide a physical barrier for the immune system, leading to exclusion of CD8^+^ T cells.^52^, ^53^ Collagen‐1 deposition has also been shown to be essential to release cells from latency.^54^ Secreted cytokines can induce pro‐survival and migration pathways in tumor cells and modulate the immune microenvironment.^49^ Thus, activation of stromal cells, mainly HSCs, by tumor cells and tumor‐stroma crosstalk is an important step to adapt the local niche to form micrometastasis.

#### 2.3.4 Steps 7 and 8: Overcoming hypoxia through angiogenesis

To overcome hypoxia induced size restriction of micrometastasis, angiogenesis is required.^55^ Interplay between the established niche and tumor cells is important to induce angiogenesis. Hypoxic cells will secrete angiogenic factors including vascular endothelial growth factor (VEGF)A. VEGFA mediates endothelial cell migration, promoting formation of new blood vessels. This could be observed in liver micrometastases containing high numbers of VEGFA‐secreting aHSCs.^56^ Indeed, aHSCs were found to mediate LSEC vessel formation *in vitro*.^57^ Recently, a crosstalk between LSECs, HSCs, and tumor cells was elucidated in the context of stroma activation and angiogenesis.^47^ ICAM‐1 expressed on LSECs promoted secretion of IL‐6, prostaglandin E2, VEGF, and MMP2 by tumor cells, which in turn induced VEGFA and MMP2 secretion by HSCs. These cytokines stimulated migration and angiogenic potential of LSECs and HSCs.^47^ In addition to aHSCs and tumor cells, neutrophils from liver metastases were also shown to express pro‐angiogenic factors including fibroblast growth factor 2 (FGF2). Depletion of FGF2 impaired vascular structure in liver metastases.^58^ Thus, a variety of cell types can aid in metastatic colonization by secreting pro‐angiogenic factors. Once new blood vessels are formed, micrometastasis can overcome their growth restraints by hypoxia and develop into macrometastases.

### 2.4 TGF‐β can mediate adaptation of tumor and stroma cells to overcome bottleneck of metastasis

The efficiency of liver colonization is dependent on the adaptation of tumor cells and the interaction between tumor cells and liver microenvironmental cells to induce stromal activation, angiogenesis, and immune suppression.^32^ While metastases will differ between patients and cancer types, processes leading to overcoming the metastatic bottleneck and establishment of liver metastasis could be shared, and therefore targeted. A key cytokine that is involved in all these processes necessary for efficient hepatic colonization is TGF‐β.^12^, ^13^ Indeed, liver metastases have been found to be dependent on TGF‐β signaling in liver stroma, while TGF‐β signaling in tumor cells promotes invasion and outgrowth.^59^, ^60^, ^61^, ^62^

## 3 TGF‐β SIGNALING

**FIGURE 2:**
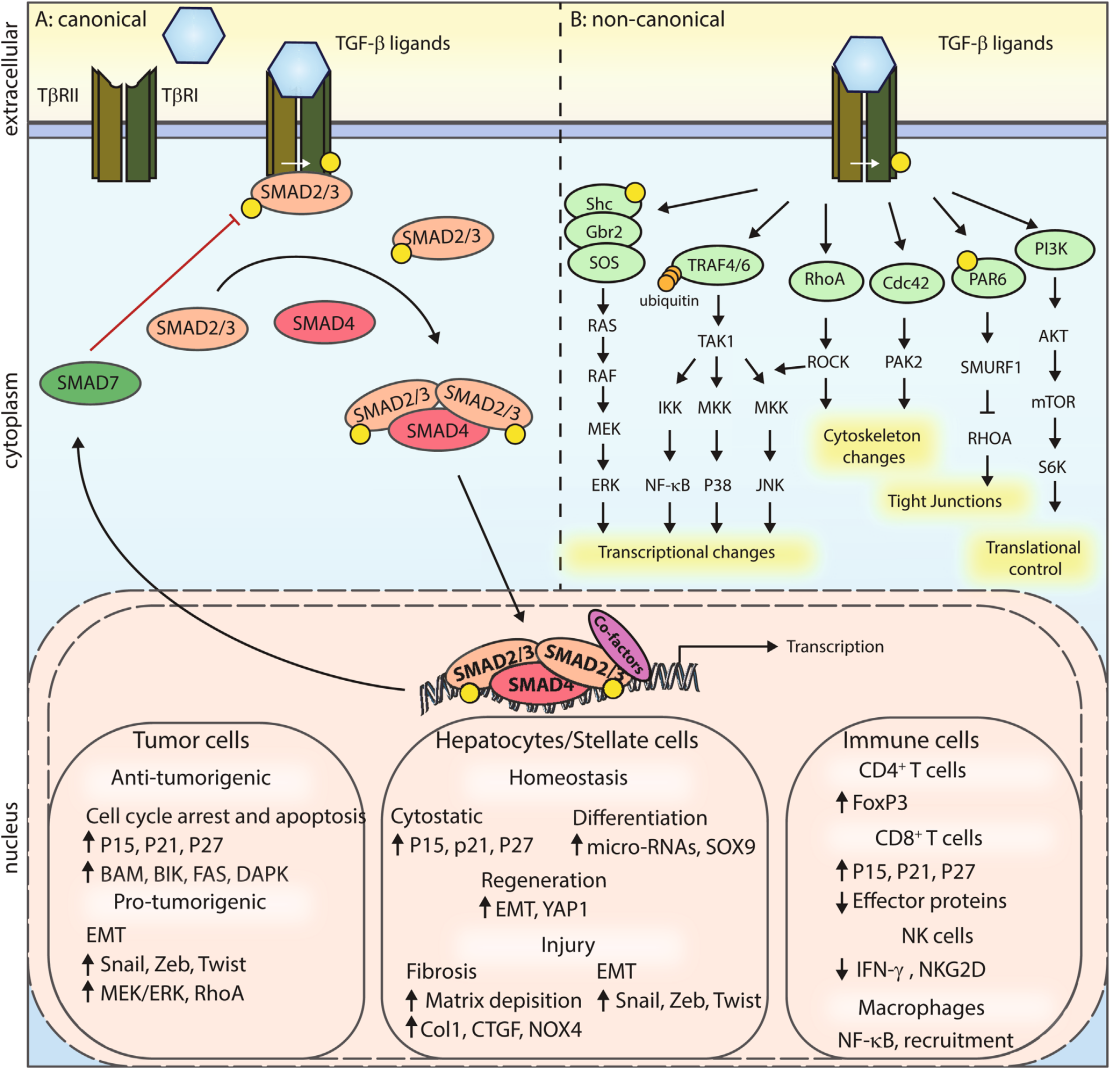
Canonical and non‐canonical TGF‐β signaling. Transforming growth factor (TGF)‐β ligands initially bind to TGF‐β receptor(TβR)II, and thereafter recruit TβRI. A heteromeric TGF‐β receptor complex is formed in which the TβRII kinase transphosphorylates TβRI. A, In canonical TGF‐β signaling, intracellular effectors mothers against decapentaplegic homolog (SMAD)2 and SMAD3 are phosphorylated by TβRI, which thereafter can bind to SMAD4. This complex translocates into the nucleus, and together with other co‐factors, regulates the expression of TGF‐β target genes in a context dependent manner. A negative feedback loop is created through induced expression of inhibitory SMAD7, which by partnering with Smad ubiquitination regulatory factor(SMURF) E3 ubiquitin ligases, targets the TβRI for ubiquitin‐mediated degradation. B, Non‐canonical TGF‐β signaling includes multiple different signaling pathways that are not unique for TGF‐β signaling, an overview is given in this figure. TGF‐β can elicit different responses in different cell types, depending on the cellular context. In tumor cells, TGF‐β/SMAD pathway can induce cytostatic effects through upregulation of cell cycle arrest and apoptosis genes. Pro‐tumorigenic effects of TGF‐β occur through SMAD‐dependent upregulation of EMT transcription factors and also non‐canonical signaling play a role. In hepatic and stellate cells, TGF‐β can induce cytostatic effects, but also differentiation and regeneration. During injury, TGF‐β signaling promotes fibrosis through e.g. matrix deposition and stimulating EMT. In immune cells, TGF‐β dampens immune responses. Cytotoxic effects of CD8^+^ T cells and natural killer (NK) cells are repressed, while regulatory CD4^+^ T cells and (immune suppressive) macrophages are promoted

TGF‐β family members play essential roles in epithelial, endothelial, tissue, and immune cells, where they regulate processes such as cellular proliferation, survival, migration, and differentiation. The TGF‐β family consists of three mammalian TGF‐β isoforms, TGF‐β1, TGF‐β2, and TGF‐β3, and structurally and functionally related proteins.^63^ All family members have multiple functions, which are highly dependent on the cellular context.^13^ Signaling of TGF‐β cytokines occurs through canonical mothers against decapentaplegic homolog (SMAD) signaling and through crosstalk with a multitude of non‐canonical signaling pathways. Figure 2 gives an overview of the canonical and non‐canonical signaling pathways induced by TGF‐β, as well as its pleiotropic effects in different cell types involved during liver metastasis formation.

### 3.1 Canonical and non‐canonical TGF‐β signaling transduction

The canonical TGF‐β signaling pathway is activated upon TGF‐β binding to two serine/threonine kinase receptors TGF‐β type II receptors (TβRII), leading to the recruitment and formation of a heteromeric complex with 2 TGF‐β type I receptors (TβRI). TβRII kinase phosphorylates TβRI, which in turn phosphorylates the SMAD proteins SMAD2 and SMAD3. This induces the assembly into heterodimeric and trimeric complexes with SMAD4, which translocate to the nucleus to regulate expression of TGF‐β target genes. SMAD3 and SMAD4 are able to bind DNA, while SMAD2 cannot bind to DNA. The DNA binding strength of SMADs is relatively weak, making the binding of the complex dependent on additional DNA binding transcription factors that partner with SMADs for specific target gene regulation.^13^ SMAD signaling induces a negative feedback loop through SMAD7, which recruits the E3 ubiquitin ligase SMAD ubiquitination regulatory factor (SMURF)2 to the activated TβRI, and thereby mediating its degradation.

Besides canonical signaling, TGF‐β can induce various non‐canonical signaling pathways, either indirectly by SMAD‐induced expression of growth factors or directly downstream of the TβRI, recently summarized by Zhang.^64^ Depending on cellular context, various non‐canonical signaling pathways can thus be activated, resulting in changes in transcription, the cytoskeleton, tight junctions, and translation. Non‐canonical pathways include, for example, Rho/Rho‐associated coiled‐coil kinase (ROCK) signaling, which promotes motility and cytoskeletal rearrangements, and phosphoinositide 3‐kinase/AKT signaling, which induces translational changes. Multiple mitogen‐activated protein kinases (MAPK) signaling can be activated, including extracellular signal‐regulated kinases (ERK), through Ras activation, and c‐Jun N‐terminal kinases (JNK) and p38, through TNF receptor‐associated factor(TRAF)6 signaling, which promotes proliferation and migration.^65^, ^66^, ^67^ The above‐mentioned pathways are important to overcome TGF‐β induced cytostatic effects and promote growth, migration, and invasion.

### 3.2 TGF‐β has a dual role in tumor cells

For normal and pre‐malignant cells, TGF‐β is a potent inducer of growth arrest and apoptosis. SMAD signaling results in G1 cell cycle arrest through inducing expression of the cyclin dependent kinases inhibitors p15Ink4, P21CIP1, and P27KIP1 and inhibition of c‐MYC.^11^ TGF‐β induced apoptosis can occur through induction of apoptotic regulators.^11^ During cancer progression, tumor cells overcome the cytostatic effects of TGF‐β signaling, by acquiring mutations in the canonical signaling components, by adaptation of TGF‐β signaling through non‐canonical signaling and by gain of function of proto‐oncogenes or loss of function of tumor suppressor genes. In particular, mutations in genes encoding TβRII and SMAD4 are frequently found in gastrointestinal cancers.^68^ Interestingly, many other cancers have elevated TGF‐β expression and display high activity of the TGF‐β pathway that is associated with worse prognosis.^68^, ^69^, ^70^, ^71^, ^72^ In later stage tumor cells, the tumor cells can remain responsive to TGF‐β, and collaborate with other pro‐oncogenic pathways promoting cancer progression. A key mechanism in this respect is that TGF‐β can induce EMT, which is a part of the dynamic epithelial‐mesenchymal plasticity of tumor cells.^73^ EMT is characterized by the loss of epithelial cell character, cell polarity, and cell‐cell junctions, and the gain of mesenchymal features, resulting in more invasive potential.^11^ Moreover, EMT contributes to chemo‐resistance and immune evasion, as summarized by Hao et al.^11^ EMT induction by TGF‐β/SMAD signaling is cell and context specific, and can be achieved by inducing the expression of, for example, Snail, Zinc finger E homeobox binding, and Twist transcription factors, together with non‐canonical signaling including ERK and RhoA.^11^

### 3.3 Role of TGF‐β in the immune system

TGF‐β plays an essential role in immune cell homeostasis and suppression.^74^ In a metastatic context, TGF‐β contributes to immune evasion through multiple mechanisms, which was recently reviewed by Batlle and Massague.^75^ In short, regulatory T cells are stimulated through SMAD‐dependent induction of the forkhead box P3 (FoxP3) transcription factor, resulting in suppressed function of effector T cells. TGF‐β affects T helper 1 cells and CD8^+^ T cells directly by blocking T helper cell differentiation and blocking T cell receptor‐mediated T cell activation.^75^ T cell numbers are reduced through induction of previously mentioned cytostatic and apoptotic pathways. Additionally, SMAD signaling suppresses T cell mediated killing effector genes.^75^ In NK cells, TGF‐β can suppress recognition by downregulation of NK‐cell activating receptors, as well as blocking effector cytokines including interferon (IFN)‐γ.^75^ Moreover, TGF‐β signaling can promote differentiation towards a pro‐tumorigenic NK cell phenotype.^76^ Tumor associated macrophages (TAMs) are important sources of TGF‐β, and result in recruitment and adhesion of additional monocytes to tumor sites. Moreover, TGF‐β could contribute to differentiation of macrophages toward a pro‐tumorigenic M2 phenotype.^75^, ^77^ Overall, TGF‐β is a strong suppressor of the immune system, acting on both innate and adaptive immune responses.

### 3.4 TGF‐β signaling in healthy and diseased liver

In the liver, TGF‐β regulates processes from liver development and regeneration to liver pathologies. In healthy liver, TGF‐β1 is the predominant isoform and is mainly expressed by Kupffer cells and stellate cells, while hepatocytes show absence of TGF‐β expression. In fibrotic conditions, TGF‐β1 levels increase significantly and are found to be expressed by most sinusoid cells.^14^ During development, specific spatiotemporal distribution of TGF‐β orchestrates structural organization of the liver and modulates cellular differentiation, through microRNAs and interplay with different signaling pathways.^15^ Liver generation is partially controlled by TGF‐β signaling and crosstalk with other pathways in hepatocytes, circumventing the cytostatic effect of TGF‐β.^15^ After injury, TGF‐β1 expression is temporarily induced, increasing phosphorylation of SMAD2 and nuclear yes‐associated protein (YAP).^78^ These changes promote an EMT‐like response, leading to transdifferentiation of hepatocytes into myofibroblasts.^78^ In contrast, in the context of failed regeneration after acute extensive liver injury, it was shown that TGF‐β secreted by local macrophages induced and propagated senescence in hepatocytes.^79^ During injury, hepatocytes and Kupffer cells can be important source of TGF‐β.^80^, ^81^ TGF‐β is a key inducer of fibrosis, activating HSCs into aHSCs and transdifferentiating hepatocytes toward myofibroblasts through an EMT‐like process.^82^ TGF‐β increased matrix deposition of HSCs through inducing connective tissue growth factor (CTGF), receptor for activated c kinase 1 and NADPH oxidase 4 signaling, and collagen‐1 secretion. Mild fibrosis could be reversed by among others upregulating quiescent genes and repressing TGF‐β target genes.^82^ In conclusion, TGF‐β regulates processes in both healthy and injured liver in a highly spatiotemporal and context dependent manner.

Fibrosis is an essential component of liver metastasis, promoting liver metastasis formation as well as predicting occurrence and relapse of liver metastasis.^83^, ^84^, ^85^ In colorectal cancers, metastases are characterized by high stroma and TGF‐β signaling, resulting in poor prognosis.^86^ There is evidence that TGF‐β is not only important in creating the fibrotic niche, but also supports immune evasion and tumor outgrowth in different phases of liver colonization, in both TGF‐β wild‐type and deficient tumors.^11^, ^59^, ^60^ Orchestrating beneficial TGF‐β signaling responses in the different cell types during the different phases of metastasis will contribute to successful outgrowth and thereby pose a potential therapeutic target.

## 4 TGF‐β IN LIVER METASTASIS

**FIGURE 3:**
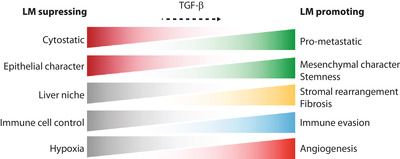
TGF‐β promotes liver metastasis at multiple angles. Transforming growth factor (TGF)‐β can influence multiple facets of liver metastases formation, promoting metastatic outgrowth. During liver metastasis, cytostatic TGF‐β signaling is suppressed in tumor cells, while pro‐metastatic signaling is promoted. Moreover, TGF‐β can promote the loss of the epithelial character and the increase of mesenchymal and stemness character of tumor cells. Through signaling in microenvironmental cells, TGF‐β can induce alterations in the liver niche to promote tumor outgrowth, through, for example, stromal rearrangement and induction of fibrosis. In immune cells, TGF‐β aids in evading immune responses. Angiogenic process promoted by TGF‐β signaling in cells promotes the influx of oxygen and nutrients in the growing liver metastasis. Abbreviation: LM, liver metastasis

Overcoming the cytostatic effects of TGF‐β is essential in metastasis formation within the liver. Mutations or deletions in genes encoding TGF‐β pathway components can be frequently found, but are not essential for all cancer types.^68^ For instance, loss of and mutations in *SMAD4* have been associated with poor prognosis and liver metastasis.^87^, ^88^ In contrast, active TGF‐β signaling and high TGF‐β plasma levels are correlated with aggressive disease, disease relapse to the liver and poor survival.^59^, ^70^, ^89^ This implies that the TGF‐β signaling output is altered (eg, by altered co‐factors or non‐canonical signaling) in aggressive tumor cells or that TGF‐β exerts tumor supporting roles in microenvironmental cells. In the liver, various cell types are a source of TGF‐β, including tumor cells, myofibroblasts, and local immune cells.^14^, ^50^, ^60^, ^90^ Below, we will describe the current knowledge of TGF‐β during the different phases of hepatic colonization. A schematic overview of different functions of TGF‐β during liver metastasis is shown in Figure 3.

### 4.1 TGF‐β signaling in liver stroma induced by the primary tumor facilitates metastatic outgrowth

**FIGURE 4:**
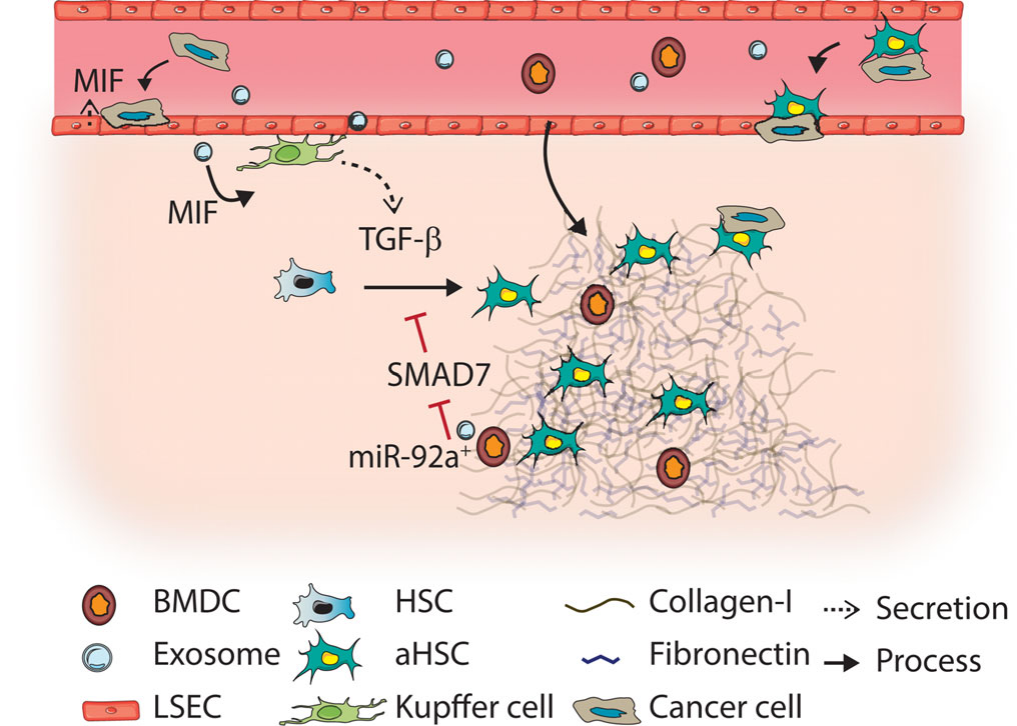
TGF‐β signaling in the pre‐metastatic niche. Primary tumors secrete cytokines and exosomes that home to the liver and trigger transforming growth factor (TGF)‐β signaling in liver cells. Exosomes containing migration inhibitory factor (MIF) triggered TGF‐β release by kupffer cells, resulting hepatic stellate cell (HSC) activation. MIF can also be secreted by liver sinusoid endothelial cells (LSECs), which increases tumor cell adhesion to LSECs. miR‐92a, present in exosomes secreted by bone marrow derived cells, represses mothers against decapentaplegic homolog(SMAD)7 in HSCs, sensitizing HSCs toward TGF‐β mediated activation. The result of TGF‐β induced HSC activation is a fibrotic niche of collagen‐1 and fibronectin, which promotes recruitment of suppressive bone marrow derived cells (BMDCs), and metastatic outgrowth. Migration of tumor cells toward the liver can be increased by TGF‐β induced interaction between tumor cells and fibroblasts

TGF‐β signaling has been linked to the creation of a permissive niche prior to arrival of tumor cells because of its key role in HSCs activation, matrix remodeling, and creation of an immune suppressive environment (Figure 4). TGF‐β signaling in liver stroma can be triggered after uptake of different cytokines or extracellular vesicles secreted by the primary tumor. For example, Costa‐Silva et al. demonstrated an essential role for PDAC‐derived exosomes in TGF‐β‐mediated pre‐metastatic niche formation.^9^ Cancer exosomes from primary PDAC cells were found to be taken up by Kupffer cells in the liver. Macrophage migration inhibitory factor (MIF) present in these exosomes stimulated TGF‐β secretion by Kupffer cells, which in turn activated HSCs leading to fibronectin and collagen‐1 deposition.^9^ This fibrotic environment increased recruitment of bone marrow‐derived macrophages and granulocytes. Upon treatment with these exosomes prior to liver metastasis induction, metastatic load was increased. Thus, MIF present in exosomes triggered crosstalk between multiple stromal cell types in the liver resulting in pre‐metastatic niche formation and increased metastatic outgrowth.^9^ Besides tumor cells, LSECs were also found to secrete MIF.^41^ *In vitro*, LSEC‐derived MIF served as a chemoattractant for CRC cells and increased primary tumor growth and metastasis formation *in vivo*.^41^ In CRC patients, primary tumor MIF levels were not correlated with distant metastasis, while higher liver MIF levels did correlate with larger liver metastasis.^41^ Overall, this supports MIF as an important pre‐metastatic niche factor that might mediate (part of) its effect through TGF‐β.

In pre‐metastatic livers of mice bearing Lewis lung carcinoma (LCC), HSCs were found to be more sensitive to TGF‐β activation through the uptake of miR‐92a in exosomes derived from bone‐marrow derived cells (BMDCs).^91^ miR‐92a targets the 3′UTR of *SMAD7*, and as a consequence inhibits SMAD7 expression, removing the negative feedback on the TGF‐β pathway. This resulted in increased aHSC and collagen‐1 in mouse livers, promoting LCC adhesion and transendothelial migration of immune suppressive granulocytic myeloid derived cells. In lung cancer patients, increased miR‐92a levels were identified in circulating extracellular vesicles, which *in vitro* indeed activated HSCs.^91^ This suggests that miR‐92 containing exosomes might sensitize HSCs to TGF‐β, which results in HSC activation and pre‐metastatic niche formation.

In conclusion, different mechanisms resulting in enhanced TGF‐β signaling in the liver have been identified for tumor cells to promote pre‐metastatic niche formation. These mechanisms are induced by exosomes secreted by tumor cells or bone marrow derived cells. The resulting fibrotic niche promotes recruitment of immune suppressing cells, attachment of disseminated tumor cells, and metastasis formation.

### 4.2 TGF‐β induced migration of tumor cells toward the liver

TGF‐β can mediate adhesion between CRC cells and isolated cancer associated fibroblasts (CAF) or endothelial cells *in vitro*, thereby potentially facilitating migration toward the liver. *In vivo*, TGF‐β positively regulated liver metastases in a tumor cell/CAF co‐injection model, possibly by enhancing co‐travelling of tumor cells and CAFs through the vasculature.^92^ Interestingly, increased adhesion to microenvironmental cells by TGF‐β was also observed in other tumor models. TGF‐β treatment of non‐invasive uveal melanoma cells increased adhesion to liver endothelial cells, promoting trans‐endothelial migration.^93^ However, this was not observed for highly invasive uveal melanoma cell lines. TGF‐β treatment showed no effect on adhesion proteins in either cell line, so the mechanism behind this specific adhesion remains unclear. Similar positive effects on transendothelial migration by TGF‐β have been observed in hepatocellular carcinoma (HCC).^94^ TGF‐β can thus promote migration towards the liver by promoting interactions with CAFs and endothelial cells.

### 4.3 TGF‐β signaling in tumor cells can increase liver metastasis formation

Deletions in genes encoding for TGF‐β components are associated with liver metastasis and poor prognosis.^68^ Moreover, canonical TGF‐β signaling in tumor cells of CRC patients suppressed liver metastasis formation.^95^, ^96^ This suggests that TGF‐β acts as a tumor suppressor in liver metastasis. In contrast, TGF‐β might act as a promoter of liver metastasis as well, as high TGF‐β signaling is correlated with poor prognosis.^68^ Indeed, pretreatment of tumor cells with TGF‐β before experimental liver metastasis formation increased the metastatic burden of a variety of cancer types.^61^, ^97^, ^98^ In another study, TGF‐β pretreatment resulted in angiogenesis, as well as tumor cells with an enhanced mesenchymal and migratory character *in vitro* and tumor cells showing increased proliferation *in vivo*. This suggests that pretreatment with TGF‐β induced changes in both tumor cells and tumor microenvironment.^61^ Moreover, knockdown of SMAD4 in CT26 CRC cells increased migration *in vitro* and liver metastasis *in vivo* through activation of, for example, TGF‐β‐induced SMAD4‐independent ERK signaling.^88^, ^89^, ^96^ These results suggest that in these cells, TGF‐β has pro‐tumorigenic effects, and TGF‐β signaling inhibition might lead to anticancer effects. Indeed, the TβRI/II small molecule kinase inhibitor galunisertib was found to block ERK‐mediated non‐canonical TGF‐β signaling in KRAS‐mutated CRC cells, inhibiting the increased migratory phenotype *in vitro* and liver metastasis *in vivo*.^62^

Besides inducing migration and EMT in tumor cells, TGF‐β can also affect the stem cell population. For example, pretreatment of orthotopically injected MDA‐MB‐231 breast cancer cells increased liver metastasis while the size of primary tumors was insignificantly affected.^99^ TGF‐β pretreatment was found to increase stem cell markers *in vitro* and slightly increase the CD44^+^CD24^−^ cancer stem cell population in liver metastases. Moreover, CD44^+^CD24^+^ cells were unable to form liver metastasis, in contrast to CD44^+^CD24^−^ cells. This suggests that a CD44^+^CD24^−^ cell population induced by TGF‐β could be responsible for increased outgrowth in the liver.^99^ Similarly, in HCC, blocking TGF‐β signaling decreased expression of CD44 and stemness features *in vitro* and *in vivo*. *Ex vivo* treatment of HCC patient samples showed reduced CD44 gene expression amongst responding patients.^100^

Combined, this suggests that based on cellular context TGF‐β signaling can have metastasis promoting or suppressive effects. The effect of TGF‐β signaling in most advanced tumor lesions is shifted towards migration, EMT or stemness, thereby promoting colonization of the liver

### 4.4 Angiogenesis can be promoted by TGF‐β signaling within the liver metastasis

**FIGURE 5:**
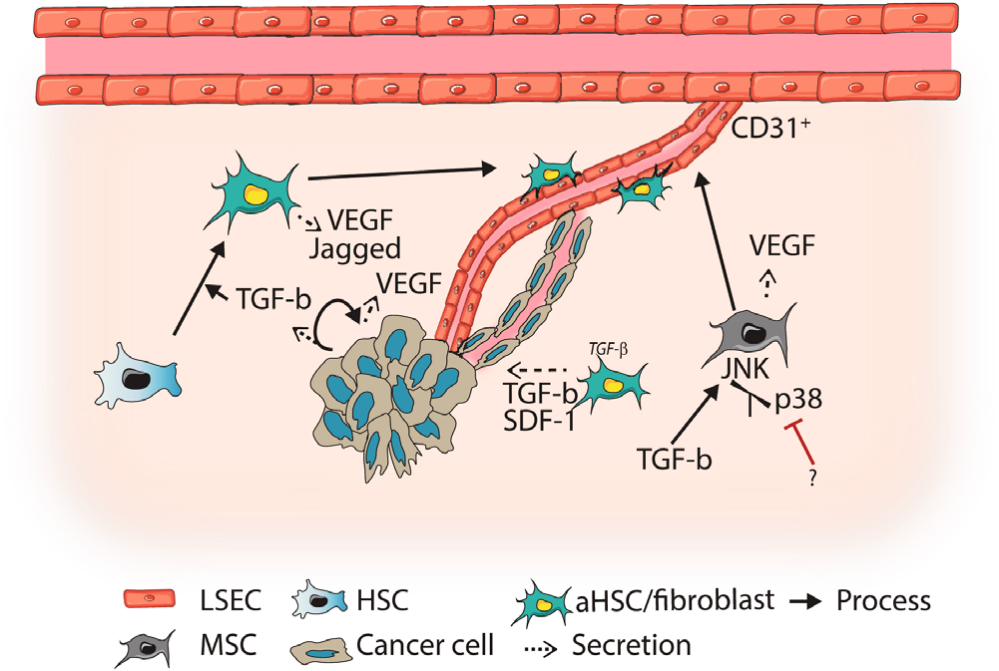
TGF‐β signaling influences angiogenic processes. Transforming growth factor (TGF)‐β signaling can elicit the secretion of angiogenic proteins, such as vascular endothelial growth factor (VEGF) and Jagged, by different cell types of the liver metastasis. This promotes (CD31^+^) vessel formation. TGF‐β and stromal derived factor (SDF)‐1 secreted by activated hepatic stellate cells (aHSCs) could promote vascular mimicry in tumor cells. Mesenchymal stem cells (MSCs) could promote angiogenesis through shifting TGF‐β responses towards c‐Jun N‐terminal kinases (JNK) signaling. This results in differentiation of MSCs into endothelial cells, and additional secretion of angiogenic factors. Abbreviation: LSEC, liver sinusoid endothelial cell

Recruitment of new blood vessels is essential for the outgrowth of micrometastases. Both non‐canonical and canonical TGF‐β signaling can influence expression of angiogenic factors, as shown in Figure 5. TGF‐β is crucial in activating HSCs, upon which they secrete various angiogenic factors like VEGF and Jagged in the surrounding matrix.^56^, ^57^, ^101^ This directly contributes to angiogenesis.^56^, ^101^ Combined, this suggests that TGF‐β can induce angiogenesis by activating HSCs, although it remains to be proven in a metastatic setting.

TGF‐β‐mediated regulation of angiogenesis is also studied in a cancer context. TGF‐β treatment increased VEGF levels in pancreatic tumor cells, and reduced tumor cell killing by peripheral blood mononuclear leukocytes. TGF‐β pretreatment also enhanced experimental liver metastasis formation. This could be due to increased angiogenesis, but remains to be proven.^102^

A new mechanism of TGF‐β‐induced angiogenesis in mesenchymal stem cells was proposed by Battle and colleages.^103^ They proposed that, upon activation of non‐canonical TGF‐β signaling, the balance between activation of P38 and JNK MAP kinases determined mesenchymal cell differentiation toward endothelial cells.^103^ P38 MAPK was found to repress angiogenic programs in mesenchymal stem cells (MSCs), while JNK MAPK signaling promoted VEGF secretion, angiogenesis, and MSC differentiation toward endothelial cells. Indeed, in an inflammation‐induced CRC model in p38αΔ^FSP1^ mice, increased CD31^+^ vessels and tumor growth were observed.^103^ Expression of these proteins are also influenced by the surrounding niche, suggesting that TGF‐β in interplay with other environmental signals can promote MSC differentiation to promote angiogenesis.

Vascular mimicry can be an alternative approach to angiogenesis, were tumor cells form channel‐like structures to mediate oxygen and nutrient influx.^104^ Interestingly, TGF‐β and SDF‐1 secretion by CAFs resulted in VM in HCC both *in vitro* and in subcutaneous tumors. This was mediated by TGF‐β‐induced ECM remodeling and expression of endothelial marker vascular endothelial VE‐cadherin by tumor cells.^104^ Likewise, increased VE‐cadherin expression by HCC cells upon TGF‐β1 treatment was also observed by Zhang et al.^105^ This resulted in VM and was dependent on TGF‐β induced EMT and Rho/ROCK2 signaling.

In conclusion, TGF‐β is able to promote angiogenesis by modulating the expression of angiogenic factors and endothelial proteins in multiple cell types, shown in Figure 5, orchestrating the formation of new blood vessels.

### 4.5 Tumor‐supportive role of TGF‐β‐activated stromal cells

In CRC patients, high TGF‐β levels are associated with relapse. Importantly, TGF‐β signaling as measured by nuclear p‐SMAD3 and TGF‐β transcriptional signatures were identified in every microenvironmental cell, and high TGF‐β signatures in CAFs predicted poor prognosis.^59^, ^86^ TGF‐β‐activated fibroblasts in another CRC model were found to secrete IL‐11, triggering GP130/STAT3 signaling in tumor cells. This repressed apoptosis, and thus enhanced colonization of the liver.^59^ This suggests that stromal TGF‐β signaling could be key in overcoming the bottleneck of metastasis.

TGF‐β‐activated stromal cells might enhance liver metastasis by the induction of a fibrotic environment.^28^ For example, in gastric cancer, TGF‐β‐activated HSCs secrete lysyl oxidase (LOX), which contributes to the fibrotic niche by crosslinking collagen fibers and increasing tumor cell proliferation through an AKT‐p70S6K‐hypoxia‐inducible factor(HIF)1‐α pathway.^106^ Besides, TGF‐β‐induced expression of miRNA‐181a in stellate cells *in vitro* was shown to promote fibrosis through inhibition of Augmenter of Liver Regeneration.^107^ The importance of a fibrotic environment for outgrowth of metastatic cells was shown by Barkan et al.^54^ TGF‐β1‐induced lung fibrosis could reactivate dormant breast tumor cells through collagen 1 and integrin β1 signaling, resulting in phosphorylation of Src, focal adhesion kinase, and ERK that led to proliferation.^54^

Stromal cell activation by TGF‐β might be enhanced by TGF‐β receptor recycling or degradation. Indeed, these processes were found altered in HSCs of CRC metastases. For example, the actin‐associated protein vasodilator‐stimulated phosphoprotein VASP mediates TβRII recycling to the cell membrane through rab11, thereby sensitizing HSCs to further TGF‐β stimulation.^108^ In addition, TGF‐β signaling in HSCs is negatively regulated by RAS GTPase activating‐like protein IQGAP1, which, by binding the TβRII, mediated SMURF‐1‐dependent ubiquitination and degradation of the receptor. In CRC patients, reduced levels of IQGAP1 were found in stroma of liver metastasis.^51^ Thus, TGF‐β receptor levels in HSCs are modulated in metastases to enhance signaling.

The source of TGF‐β that activates HSCs is often the tumor cell. For example, SDF‐1 secretion by aHSCs was found to bind C‐X‐C chemokine receptor type 4 (CXCR4), promoting TGF‐β signaling and secretion by CRC tumor cells.^50^ Blocking CXCR4 or TGF‐β *in vivo* decreased the metastatic potential of CRC cell lines and reduced HSC activation.^50^ Calon and colleagues tested the effect of TGF‐β secretion by TGF‐β‐deficient CRC cells, inducing stromal TGF‐β signaling, during liver metastasis formation in mice.^59^ Secretion of TGF‐β was found to promote both tumor initiation and metastatic potential. Interestingly, high secretion of TGF‐β1 by the CRC cells, and thus stromal TGF‐β signaling, was essential at the initial phase of liver metastasis, since a 24 hour  induction of TGF‐β secretion directly after splenic injection was sufficient to increase liver colonization.^59^ Additionally, isolated CRC stem cells from patients were found to express higher levels of TGF‐β.^59^ Thus, a feed forward loop can exist between aHSCs and tumors cells, whereby the HSCs stimulate tumor cells to secrete TGF‐β, which can activate HSCs, ultimately leading to enhanced liver metastasis formation.

At last, hepatocytes are also source of liver fibrosis next to HSCs. Lineage tracing in a CCL4 liver fibrosis model showed that hepatocytes contribute to fibroblast‐specific protein (FSP)1^+^ fibroblasts in fibrotic tissue.^109^ This was mediated by TGF‐β‐promoted EMT in hepatocytes, a process that could be antagonized by TGF‐β family member Bone Morphogenetic Protein (BMP) 7.^109^

**FIGURE 6:**
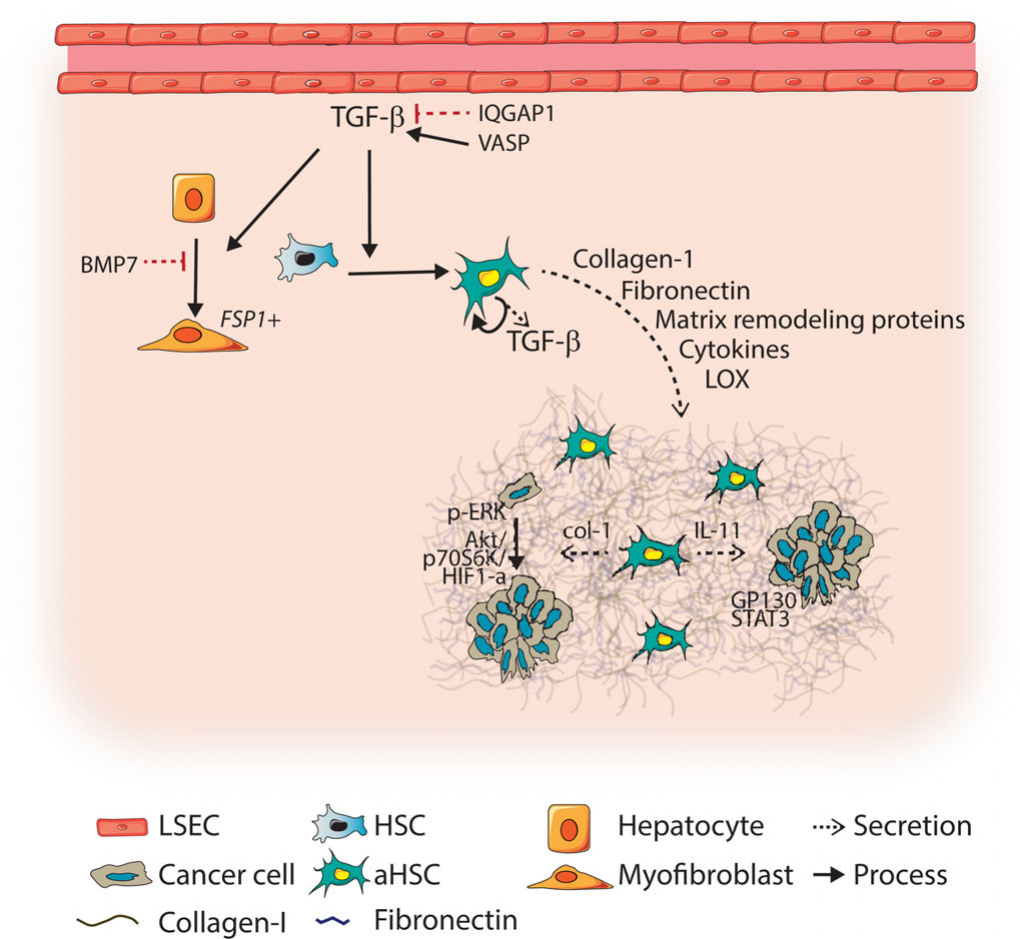
TGF‐β signaling in liver fibroblasts stimulates fibrotic environment. Transforming growth factor (TGF)‐β signaling in tumor stroma is essential for metastatic outgrowth in the liver. TGF‐β in the liver metastasis, secreted by various cell types in the metastases, promotes transdifferentiation of hepatocytes and hepatic stellate cells(HSCs) toward myofibroblasts and activated HSCs (aHSCs). In HSCs, vasodilator‐stimulated phosphoprotein (VASP) can sensitize HSCs toward TGF‐β signaling, while IQ motif containing GTPase activating protein 1(IQGAP1) represses TGF‐β signaling in HSCs. Activation of HSCs results in secretion of collagen‐1, fibronectin, matrix remodeling proteins, cytokines, and lysyl oxidase (LOX), triggering the formation of the fibrotic niche. Tumor cells in a fibrotic niche gain survival advantages through collagen‐1 induced extracellular signal‐regulated kinases (ERK) phosphorylation, which promotes proliferation. Moreover, LOX‐mediated crosslinking of collagen‐1 also increases proliferation through AKT‐p70S6K‐hypoxia‐inducible factor(HIF)1‐α signaling. Secretion of interleukin (IL)‐11 by aHSCs promote glycoprotein (GP)130/ signal transducer and activator of transcription (STAT3) signaling in tumor cells, thereby repressing apoptosis and promoting metastatic outgrowth. Abbreviations: HC, hepatocyte; LSEC, liver sinusoid endothelial cell

In conclusion, during metastasis formation TGF‐β plays a significant role in promoting metastatic outgrowth by activating stromal cells and hepatocytes and promoting matrix remodeling and fibrosis. An overview of these processes is shown in Figure 6.

### 4.6 TGF‐β plays central role in regulating an immune‐suppressive metastatic niche

**FIGURE 7:**
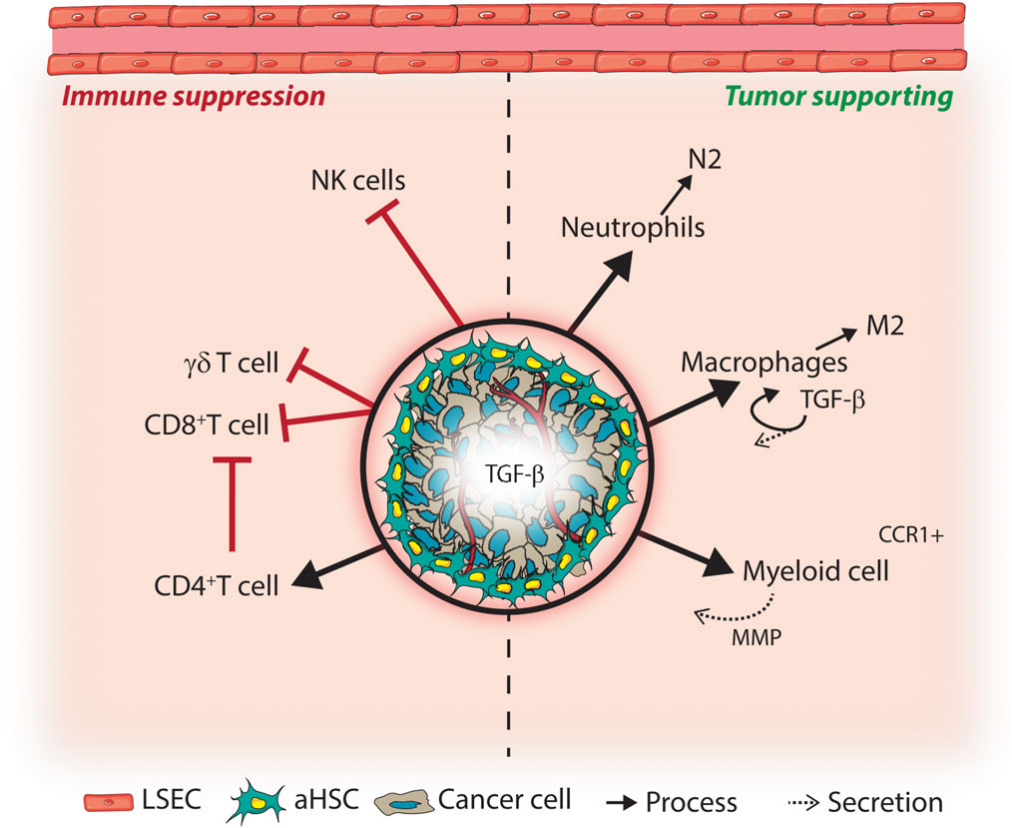
TGF‐β secretion and signaling in liver metastases can provide protection to innate and adoptive immune responses. Transforming growth factor (TGF)‐β secretion of tumor cells and fibroblasts, and the resulting fibrotic niche, provides a physical barrier against immune cells and triggers immune suppressive effects in immune cells. Neutrophils and macrophages could differentiate toward tumor supportive N2 and M2 type cells. Moreover, TGF‐β secretion by macrophages further enhance immune suppression. Anti‐tumor effects of Natural Killer (NK) cells can be inhibited by TGF‐β. Tumor supporting myeloid cells derived from the bone marrow support metastasis formation and are recruited to metastases through e.g. binding of secreted C‐C Motif Chemokine Ligand 15 (CCL15) to the C‐C chemokine receptor type 1 (CCR1) receptor. These cells support metastasis by contributing to immune suppression and secreting matrix modulating proteins such as matrix metalloproteases (MMPs). During liver metastasis formation, the effects of cytotoxic CD8^+^ and γδT cells are inhibited by TGF‐β, through e.g. reduction of T cell infiltration or proliferation, reduced T cell activation and inhibition of cytotoxic effector proteins, while immune suppressive CD4^+^ regulatory T cells are stimulated

TGF‐β has a pronounced immune suppressive effect affecting multiple immune cell populations, which is depicted in Figure 7.^75^ Therefore, TGF‐β is proposed to create immune permissive (metastatic) niches. *In vivo* studies of TGF‐β influencing the liver metastatic niche and outgrowth are limited due to the bias toward the use of immune suppressed or deficient mouse models. In light of immunotherapy, *in vivo* experiments that address the roles of TGF‐β will most likely move toward (humanized) immune competent models.

The first line of defense in the liver are innate immune cells, including NK cells, neutrophils, and macrophages. In an intravenously injected B16 melanoma metastasis model liver metastases were only observed after depletion of NK cells. TGF‐β is known to suppress NK cells at multiple levels, suggesting that TGF‐β mediated NK cell suppression could positively affect liver metastasis formation.^75^ Like metastases at other sites, metastases in the liver TGF‐β potentially induce differentiation of neutrophils and macrophages toward tumor promoting phenotypes N2 and M2, respectively.^75^, ^77^ At last, M2 macrophages produce TGF‐β, further contributing to immune suppression via a feed‐forward loop.^75^ Thus, induced TGF‐β signaling in innate immune cells can promote an immune suppressed environment.

Creating an immune suppressive niche is essential for metastatic outgrowth. Recruitment of tumor promoting BMDCs contributes to this process. In addition, BMDCs are important in promoting invasion and metastasis through MMP secretion.^110^ In experimental metastasis models of SMAD4‐deficient compared to SMAD4‐proficient CRC, BMDCs were recruited to liver metastases through increased secretion of C‐C motif chemokine ligand (CCL)9 (mouse ortholog of CCL15), the ligand for the C‐C chemokine receptor type 1 (CCR1) receptor on myeloid cells. *In vitro*, it was found that SMAD4 binds to the promotor of CCL15 to inhibit CCL15 expression.^111^ The correlation between CCL15^+^ tumor cells and CCR1^+^ BDMCs was also observed in CRC liver metastasis of patients.^110^ Thus, loss of SMAD4 during liver metastasis promotes secretion of CCL15 and recruitment of immune suppressive BMDCs.

Evading adaptive immune responses is another essential characteristic for successful metastasis formation, and exclusion of T cells is observed in growing metastases.^3^, ^53^ Also in C26 CRC liver metastases compared to wildtype livers, a decrease in CD8^+^ T cells was observed livers, while the population of T regulatory cells, suppressing immune responses, was increased.^112^ This shift was accompanied by increased expression of IL‐10 and TGF‐β in liver tissue containing metastases. Similarly, in HCC, decreased numbers of γδ T cells were observed intratumorally compared to peritumorally, as well as reduced IFN‐γ and granzymes secretion exerting tumor cytotoxicity.^113^ Ex vivo, this was dependent on CD4^+^ regulatory T cells, and IL‐10 and TGF‐β secretion.^113^ In pancreatic cancer, ICAM‐1 cell surface levels were reduced after TGF‐β treatment *in vitro*. ICAM‐1, expressed by T cells, serves as a ligand for integrin β2 and results in T cell activation.^114^ These studies suggest that TGF‐β mediates tumor cell evasion of the adaptive immune response, and that blocking TGF‐β would result in tumor cell killing. Indeed, Medina‐Echeverz and colleagues reported increased T cell‐mediated killing and IFN‐γ production in MC38 CRC and metastasizing melanoma mouse models upon treatment with high density lipoproteins containing the TGF‐β blocking peptide P144.^115^

More recently, Tauriello et al. used an elegant genetically engineered CRC model to demonstrate the strong immune evasive effect of TGF‐β during liver metastasis.^60^ Using a quadruple mutant (including TβRII) CRC organoid model, liver metastases were formed after implanting mouse tumor organoids in the caecum. These liver metastases were characterized by high stromal TGF‐β signaling and low T cell infiltration, reflecting a poor prognosis subtype of CRC. Blocking TGF‐β signaling in CAFs and T cells through galunisertib significantly reduced metastasis formation, mainly by enhancing T cell‐mediated tumor cell killing in early metastatic stages.^60^ However, in established metastatic disease galunisertib resulted in mild effects. This was attributed to acquired resistance to T cell‐mediated killing due to increased expression of PD1/PDL‐1.^60^ Indeed, combinational treatment of galunisertib and anti‐PD‐L1 antibodies significantly increased efficiency of the therapy.

In sum, studies in the liver suggest that, like in other organs, TGF‐β plays an important role in immune suppression at the innate and adaptive immune response level, as shown in Figure 7. This provides promise to TGF‐β inhibitors to treat metastatic liver disease.^116^ This avenue of research could greatly improve response to immunotherapy and metastatic cancer patient survival.

## 5 IMPROVING CURRENT CLINICAL STRATEGIES

TGF‐β is important in creating the fibrotic niche and angiogenesis, and it supports immune evasion and tumor outgrowth in different phases of liver colonization, in both in TGF‐β wild‐type and deficient tumors. Orchestrating metastasis promoting TGF‐β signaling responses in the various liver niche cell types during the different phases of metastasis contributes to successful outgrowth. Thus, TGFβ poses an interesting potential therapeutic target.

The main treatment aiming for long‐term survival for patients with liver metastasis is surgery. Unfortunately, few patients qualify for surgery and additionally, surgery triggers fibrotic processes that could enhance the chance of recurrence.^25^ Considering the effect of TGF‐β in all essential processes in cancer and metastasis formation, TGF‐β targeting therapies could pose a treatment target as monotherapy or in combinational therapy. A mouse model engineered to secrete a soluble TGF‐β antagonist showed protection against metastasis in, among others, the liver.^117^ Several TGF‐β targeted therapies including receptor kinase inhibitors, small molecules, neutralizing antibodies and TGF‐β ligand traps, have been developed and are tested in (pre)clinical studies, as monotherapy or combinatorial treatment.^118^

TGF‐β has pleiotropic effects, and as such, targeting TGF‐β might potentially lead to on‐target side effects. Indeed, the main adverse effects observed with TGF‐β targeting therapies are related to cardiovascular toxicity, and hyperproliferation due to blocking of the antiproliferative function of TGF‐β during tissue homeostasis.^12^ Clinical trials on TGF‐β targeted drugs should carefully examine these on‐target side effects. Moreover, careful considerations on treatment schemes and doses (eg, drug holidays) are warranted. Importantly, the limited toxicity and side‐effects seen in a clinical trial with Galunisertib holds great promise for TGF‐β inhibition therapy.^119^

### 5.1 TGF‐β inhibitors in (pre)clinical studies

TGFβRI/II kinase inhibitor Galunisertib is currently studied in phase II clinical trials. This drug showed moderate benefit for unresectable pancreatic cancer patients in combination with Gemcitabine, while also showing limited toxicity.^119^ In contrast, a phase II clinical trials in glioblastoma patients did not find beneficial effects of galunisertib as monotherapy or in combination with Lomustine.^121^ In HCC, galunisertib was shown to decrease stemness of invasive HCC cells *in vitro*, as well as in *ex vivo* samples of HCC patients responding to Galunisertib.^100^ TGF‐β also plays an important role during fibrosis, therefore Hammad et al. studied the use of galunisertib in liver fibrosis.^122^ In *Abcb4Ko* mice modeling chronic liver fibrosis, treatment with galunisertib did not reduce fibrosis, but did reduce the mRNA levels of fibrotic genes and levels of laminin‐332, and β‐catenin. Similar effects of galunisertib were seen in a human ex vivo liver fibrosis model.^123^ These results indicate that inhibiting TGF‐β might not reverse fibrosis, but could remodel the matrix and potentially dysregulate stroma crosstalk in a metastatic setting.^122^ Overall, galunisertib seems safe and shows some beneficial response as a monotherapy to treat metastases.

The monoclonal antibody fresolimumab neutralizing TGF‐β1, 2, and 3 showed modest effects in patients in a small phase 1 clinical trial of patients with malignant melanoma or renal carcinoma.^124^ In metastatic breast cancer, the combination of fresolimumab with focal radiation was explored. Radiation evokes a damage response in tissues triggering, for example, TGF‐β signaling, therefore, combinatorial treatment with TGF‐β inhibitors could potentially be beneficial. Combination of radiation with higher doses of fresolimumab increased median overall survival compared to lower dosing, although with moderate response.^125^ Interestingly, higher dose of fresolimumab showed increased levels of e.g. memory CD8^+^ T cells.^125^

Thus, many TGF‐β inhibitory strategies show promising results in preclinical studies, however, the use of TGF‐β targeted therapy in the clinic has only shown moderate to small effects on the metastatic burden and proves to be challenging. As such, combinatorial treatment is more likely to be effective.

### 5.2 Combining TGF‐β inhibitors and immunotherapy

Immunotherapy is an extremely promising therapy showing great benefit for responding patients. Currently, the main immunotherapeutic drugs are the immune checkpoint inhibitors anti‐cytotoxic T lymphocyte antigen 4 antibody ipilimumab, and antibodies targeting programmed cell death 1 (PD1), pembrolizumab, and nivolumab.^126^ Despite the long‐lasting, and sometimes curative responses in some patients, the percentage of responders is low. A meta‐analysis by Li et al. showed that patients with liver metastases of various cancer types treated with anti‐PD1/PD‐L1 had a better prognosis compared to conventional treatment. However, CD8^+^ T cell counts in the liver were lower compared to non‐liver metastatic sites, which might contribute to the higher immune tolerance of the liver.^127^, ^128^ Therefore, combinational therapies targeting the immune system might be of added value specifically in the liver. As TGF‐β shows a clear immune suppressive role, inhibition of TGF‐β might elevate this suppression, and combining this inhibition with immunotherapy might improve the response rate of immunotherapy. Additionally, TGF‐β‐induced EMT correlated with increased PD‐L1 levels through microRNA‐200/ZEB1 signaling, giving an additional reason for tumors to be more responsive to anti‐PD‐L1 treatments.^129^

The potential of combining TGF‐β inhibition with immunotherapy targeting PD‐L1 was shown in studies performed by Tauriello and by Mariathasan.^60^, ^116^, ^130^ Looking at a large phase 2 trial of metastatic urothelial cancer treated with PD‐L1 inhibitors, non‐responders were characterized by increased TGF‐β signaling in fibroblasts, mediating T cell exclusion. Combinatorial treatment induced stromal remodeling, decreased TGF‐β signaling and increased influx of CD8^+^ T cells.^130^ Similarly, galunisertib treatment increased sensitivity to anti‐PD‐L1 treatment in a CRC mouse tumor organoid model, as TGF‐β targeting treatment increased stromal PD‐L1 and T cell programmed cell death (PD)‐1 expression.^60^ Dual TGF‐β and PD‐L1 inhibition showed great synergistic effects on tumor regression in these models.^60^, ^130^ Similarly, TβRI inhibition together with anti‐CTLA‐4 treatment resulted in synergistic effects on tumor growth in a melanoma mouse model.^131^ Interestingly, in this model, TGF‐β inhibition led to increased CAF proliferation and to reduced PD‐L1 surface expression on tumor cells through MMP9‐mediated cleavage, suggesting a resistance mechanism for anti‐PD1 therapy.^131^ This could be circumvented by inhibiting TβRI 3 weeks after the start of PD‐1 inhibition, showing that there is room to explore optimal combination treatment conditions.^131^

New studies of combinatorial treatment with TGF‐β inhibitors and immunotherapy are now emerging.^131^, ^132^, ^133^, ^134^ Bintrafusp alfa, a PD‐L1 IgG fused to two extracellular domains of TβRII molecules connected by peptide linkers was developed by Lan and colleagues.^120^ This peptide traps TGF−β molecules and targets PD‐L1. In multiple syngeneic mouse models, this drug showed enhanced response compared to treatment with anti‐PD‐L or TGF‐β trap alone. Moreover, bintrafusp alfa reduced the development of (lung) metastasis in spontaneously metastasizing orthotopic breast cancer models.^120^, ^135^ Compared to anti‐PD‐L1 treatment alone, monotreatment with bintrafusp alfa increased the tumor density and activity of CD8^+^ T cells, NK cells, neutrophils, and tumor associated dendritic cell and M1 macrophages.^120^, ^135^ Interestingly, myofibroblasts also seemed to be affected by bintrafusp α, since a decrease in α‐SMA was seen. When bintrafusp alfa was combined with additional CXCR1/2‐IL8 inhibition, EMT related resistance mechanisms to checkpoint inhibitors were overcome, leading to an additional synergistic effect.^136^ The first phase I clinical trials with bintrafusp alfa have been completed, showing tolerance and initial therapeutic benefit in various cancer types.^135^ However, larger clinical trials are necessary to draw firm conclusions. Mouse models have shown the added benefit of bintrafusp alfa with radiation and chemotherapy, providing directions for potential use for bintrafusp alfa in the clinic.^120^

Studies combining TGF‐β targeting therapies with immunotherapies are still in early stages, but aforementioned results clearly show the promises of this approach.^137^

## 6 CONCLUSION AND FUTURE PERSPECTIVE

As an abundant cytokine with pleiotropic functions, TGF‐β has been shown to play important roles in multiple aspects of liver metastasis formation, as illustrated in Figure 3. Creating optimal growth conditions and adaptation at distant sites is essential for metastatic outgrowth. Hitting multiple aspects of this process therefore poses as a promising strategy for eliminating liver metastasis formation and preventing cancer‐related deaths. This review has illustrated that TGF‐β can be involved in all these individual processes. Tumor cells modulate TGF‐β signaling to their advantage, promoting EMT, motility, and therapy resistance mechanisms. Within the liver stroma, TGF‐β signaling in multiple cell types orchestrates a tumor supportive environment which is necessary to survive, grow, attract blood vessels, and shield metastases. Additionally, the importance of the immune suppressive function of TGF‐β in metastasis is becoming clear. Even though TGF‐β will most likely not be the sole regulator of any of these processes, the multiplicity of targets makes TGF‐β inhibition treatment an extremely attractive approach for many different cancer types. In patients, TGF‐β inhibitor responses might be heterogeneous due to mutational signatures (in TGF‐β components) and crosstalk with non‐canonical TGF‐β signaling. However, even if tumor cells remain unresponsive to TGF‐β, effects on the microenvironment still remain. Moreover, the strength of anti‐TGF‐β treatment will probably lay in combination therapy, since TGF‐β inhibitor treatment alone showed moderate responses. By combining anti‐TGF‐β treatment with immunotherapy, not only will the effect of immunotherapy be enhanced, potential mechanisms of resistance, fibrosis, and angiogenesis might also be affected, intensifying the anti‐cancer effect together with response rates of these drugs. Similarly, damage‐induced fibrosis due to chemotherapy or surgery might be dampened by this approach. Combinatorial therapy approaches will most likely be a focus of future research, to target the complexity and dynamics of metastases. One needs to keep in mind the pleiotropic nature of TGF‐β. On‐target side effects can be expected and are reported, so dosing (eg, through drug holidays) and toxicity should be carefully studied in order to safely apply these approaches in the clinic. Specific drugs delivered to for example aHSCs could help reduce the toxicity of TGF‐β targeted drugs.^138^ Moreover, since the liver has essential roles in metabolism and waste removal, it is possible that TGF‐β targeting drugs accumulate in the liver, allowing for the use of lower systemic drug concentrations. This could make the liver specifically suitable for TGF‐β targeted therapies.

Current studies investigating the role of TGF‐β signaling in liver metastasis only provide a snapshot into metastases. However, cancer cells are not static but instead display dynamics and plasticity of processes, such as epithelial to mesenchymal plasticity. Considering the pleiotropic and dynamic nature of TGF‐β signaling, future studies should focus on unraveling these dynamics to gain better understanding on how and when to target TGF‐β signaling. Intravital imaging approaches in (immunocompetent) mice could advance this field of study.^139^, ^140^

## AUTHOR CONTRIBUTIONS

DLM and LR initiated and designed the review. DLM wrote the manuscript. LR supervised the study. LR, PtD, and RH corrected the manuscript. RH performed a feasibility study and identified key references. All authors approved the final version.

## DECLARATIONS

This work is part of the research program 016.176.081 (Veni, L. Ritsma), which is financed by the Dutch Organization for Scientific Research (NWO). Also, we would like to thank the financial support of the LUMC Gisela Their Fellowship grant, and a subsidy from the Leids Universiteits fonds (LUF, CWB 7204, L. Ritsma), and subsidies from the Cancer Genomics consortium (L. Ritsma and P. ten Dijke).

## AUTHORS’ INFORMATION

D.L. Marvin is a PhD student under the supervision of Dr. L. Ritsma and Prof. P. ten Dijke. All three have an interest in studying the dynamics of TGF‐β signaling during metastatic colonization, particularly in the liver. Prof. ten Dijke is an expert in TGF‐β signaling. Dr. Ritsma is an expert in advanced microscopy techniques. R. Heijboer wrote her Masters’ thesis on the subject of TGF‐β signaling in liver metastasis under the guidance of D.L. Marvin.

## CONFLICT OF INTEREST

The authors declare no conflict of interest.
